# Supplementary material for: GPNMB marks a quiescent cell population in melanoma and promotes metastasis formation
Source: EMBO Rep. 2025 Jun 17;26(15):3804–30. doi: 10.1038/s44319-025-00501-w (PMC12332017; doi:10.1038/s44319-025-00501-w)
Supplement: Supplementary file 1 — Appendix [file 44319_2025_501_MOESM1_ESM.pdf]

APPENDIX

Table of content	
Appendix Figure S1 and Legend	p. 1-2
Appendix Figure S2 and Legend	p. 3
Appendix Figure S3 and Legend	p. 4
Appendix Figure S4 and Legend	p. 5-6
Appendix Figure S5 and Legend	p. 7-8
Appendix Figure S6 and Legend	p. 9-10
Appendix Figure S7 and Legend	p. 11
Appendix Figure S8 and Legend	p. 12
Appendix Figure S9 and Legend	p. 13-14
Appendix Table S1	p. 15
Appendix Table S2	p. 16
Appendix Table S3	p. 17

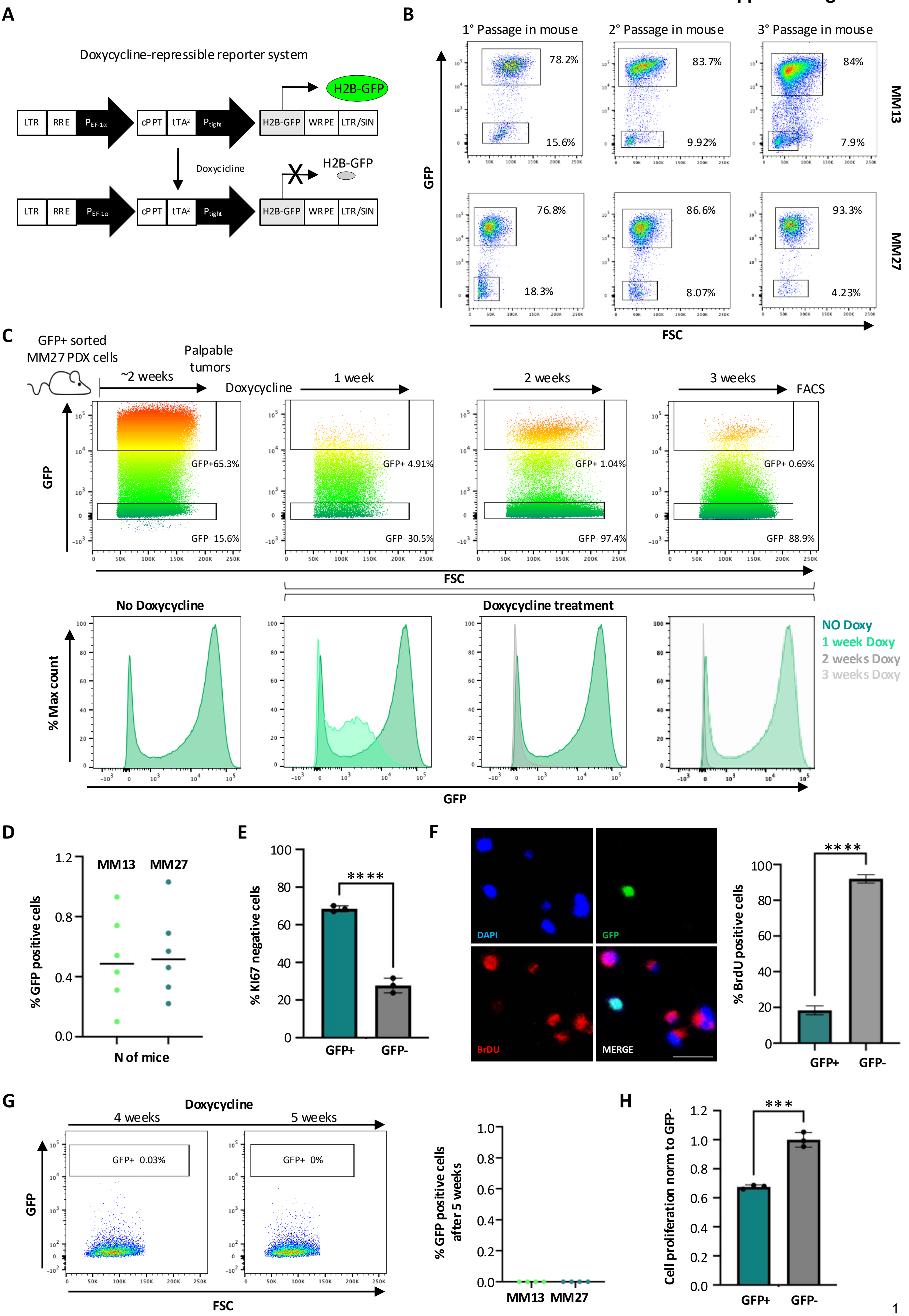
A
Doxycycline-repressible reporter system
LTR
RRE
PEF-1α
cPPT
tTA2
Ptight
H2B-GFP
WRPE
LTR/SIN
Doxycicline
B
1° Passage in mouse
2° Passage in mouse
3° Passage in mouse
78.2%
15.6%
83.7%
9.92%
84%
7.9%
MM13
76.8%
18.3%
86.6%
8.07%
93.3%
4.23%
MM27
GFP
FSC
C
GFP+ sorted MM27 PDX cells
~2 weeks
Palpable tumors
Doxycycline
1 week
2 weeks
3 weeks
FACS
GFP+65.3%
GFP- 15.6%
GFP+ 4.91%
GFP- 30.5%
GFP+ 1.04%
GFP- 97.4%
GFP+ 0.69%
GFP- 88.9%
No Doxycycline
Doxycycline treatment
% Max count
NO Doxy
1 week Doxy
2 weeks Doxy
3 weeks Doxy
D
MM13
MM27
% GFP positive cells
N of mice
E
% KI67 negative cells
****
GFP+
GFP-
F
DAPI
GFP
BrDU
MERGE
% BrdU positive cells
****
G
Doxycycline
4 weeks
5 weeks
GFP+ 0.03%
GFP+ 0%
% GFP positive cells after 5 weeks
MM13
MM27
H
Cell proliferation norm to GFP-
***

Appendix Figure S1. Isolation of label-retaining MM27 PDX cells *in vivo* by H2B-GFP tet-off vector expression.

A Scheme of the doxycycline-repressible histone H2B-GFP reporter system, stably incorporated into the DNA, constructed by combining Tet-Off regulatory elements with the Histone 2B-GFP (H2B-GFP) fusion construct into a lentiviral HIV-1 based vector backbone. $P_{EF\text{-}1\alpha}$, elongation factor 1α promoter; tTA2 and Ptight, Tet-trans-activator and promoter; LTR/SIN, self-inactivating retroviral long terminal repeat sequences; RRE, HIV Rev response element; cPPT, central polypurine tract; WPRE, *Woodchuck* hepatitis virus post-transcriptional regulation element. **B** GFP+ MM13 (upper panel) and MM27 (lower panel) cells were serially passaged ($2x10^5$ cells/passage injected intradermically) *in vivo* in NSG mice to purify the GFP+ population. Representative flow cytometry analysis of GFP+ and GFP- sorted populations over passages are shown. **C** H2B-GFP transduced MM27 cells chased *in vivo* with doxycycline. GFP-expressing, FACS-sorted MM27 PDX cells ($2x10^5$) injected in NSG mice grown for 2 weeks and then treated with doxycycline. Representative dot-plot flow cytometry analysis (upper panel) and distribution histograms of GFP expressing cells (lower panel) show cell proliferation (gradual GFP loss) over time with around 0.5% of the cells retaining GFP label 3 weeks after treatment. **D** Percentage of MM13 and MM27 GFP+ cells isolated by cytofluorimetry after 3 weeks of doxycycline treatment (n=6 biological replicates mice/PDX). **E** MM27 GFP+ and GFP- cells, after 3 weeks of doxycycline treatment, were stained with KI67 and analyzed by flow cytometry. Data are presented as mean ± SD (n=3 biological replicates). Student t test was used (*****P*=7.33e-5). **F** MM27 GFP+ and GFP- cells, after 3 weeks of doxycycline treatment, were pulsed for 24h with BrdU *in vitro* and chased for 24h. Cells were analyzed by confocal microscopy (representative images) and counted for GFP and BrdU expression by ImageJ. Data are presented as mean ± SD (n=5 technical replicates). Student t test was applied to assess the significance (*****P*=3.98e-11). Scale bar = 200µm. **G** Representative dot-plot flow cytometry analysis (left panel) and histograms (right panel) of MM27 H2B-GFP-transduced cells treated with Doxycline for 4 and 5 weeks *in vivo*. GFP expression is lost 5 weeks after treatment. **H** MM27 GFP+ cell proliferation, after 3 weeks of doxycycline treatment, was assessed by CyQuant over GFP- as mean ± SD (n=3 technical replicates). p-values are based on unpaired Student's t test (****P* =0.0004).

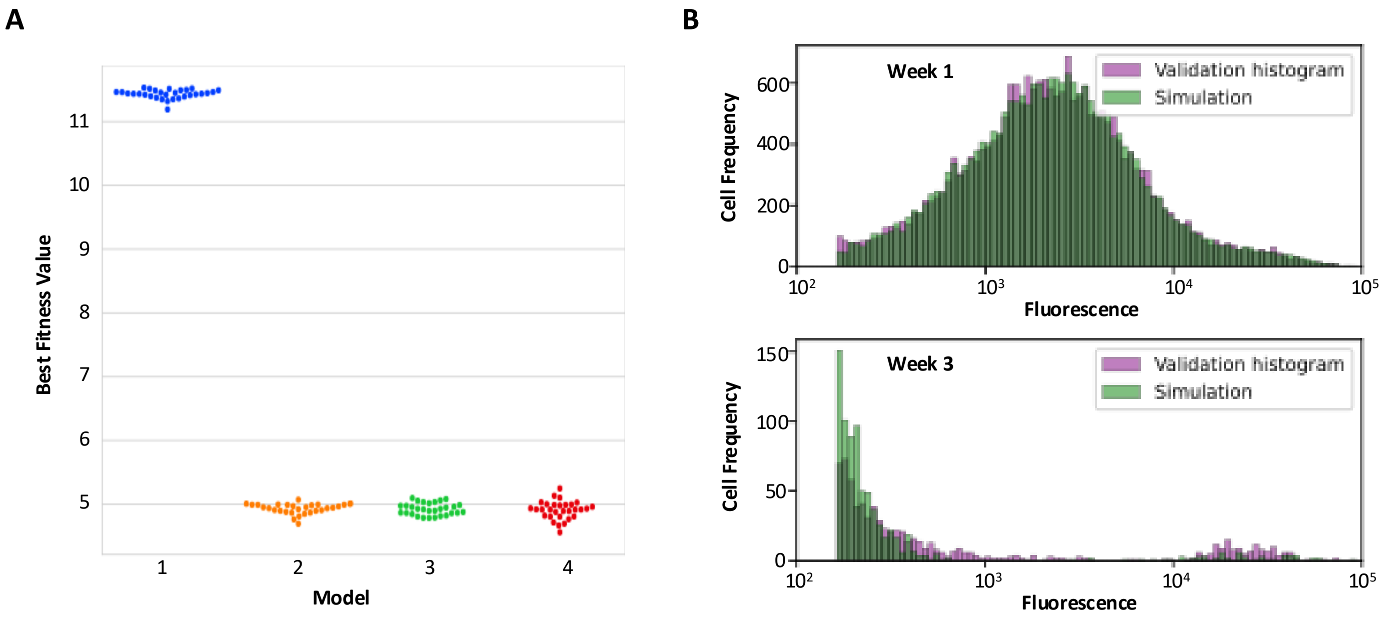

Appendix Figure S2. ***In silico*** **modeling and simulation of cell proliferation.**

A Swarm plot of the fitness of 4 *in silico* cell proliferation models. **B** Models validation using best parameterization and simulating the various configurations 100 times each, at week 1 (168 h, upper panel) and at week 3 (504 h, lower panel).

A

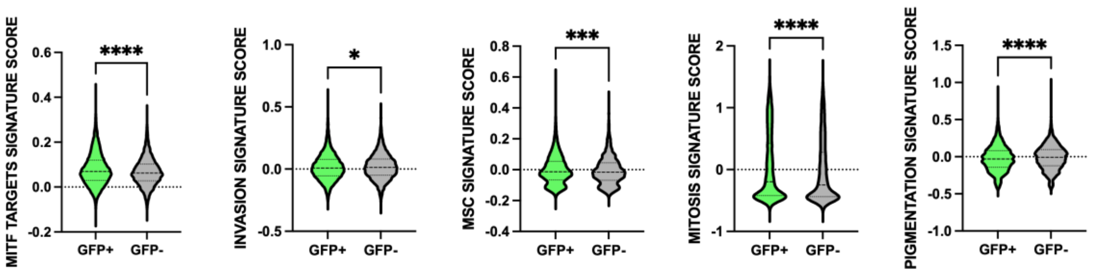

B

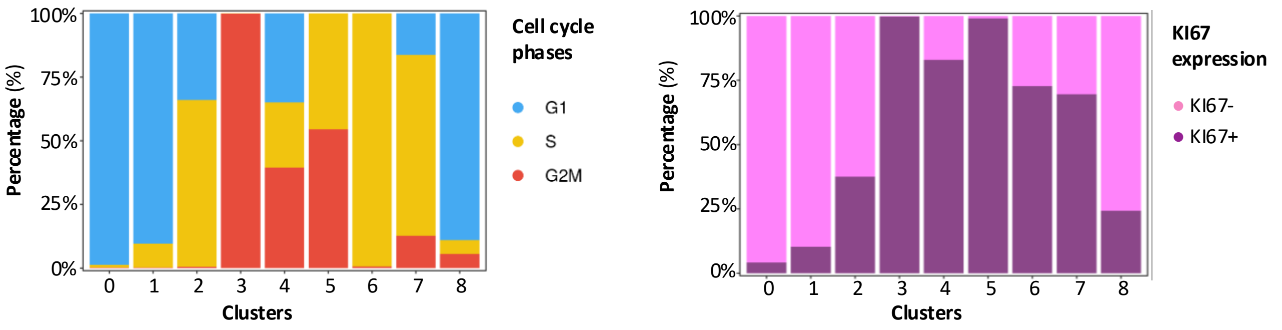

Appendix Figure S3. Analyses of published melanoma signatures in GFP+ and GFP- MM13 populations.

A Violin plot illustrating the expression of melanoma signatures from Rambow et al (12) in GFP+ and GFP- MM13 cells. p-values were determined by Mann-Whitney U-test (* P=0.01; *** P=0.0008; ****P=2.44e-14; 3.02e-13; 6.89e-10). **B** Stack plots showing relative proportions of MM13 melanoma cells annotated according to G1, S, G2/M cell cycle phases (left) and KI67 expression (right) across Seurat clusters.

Appendix Figure S4

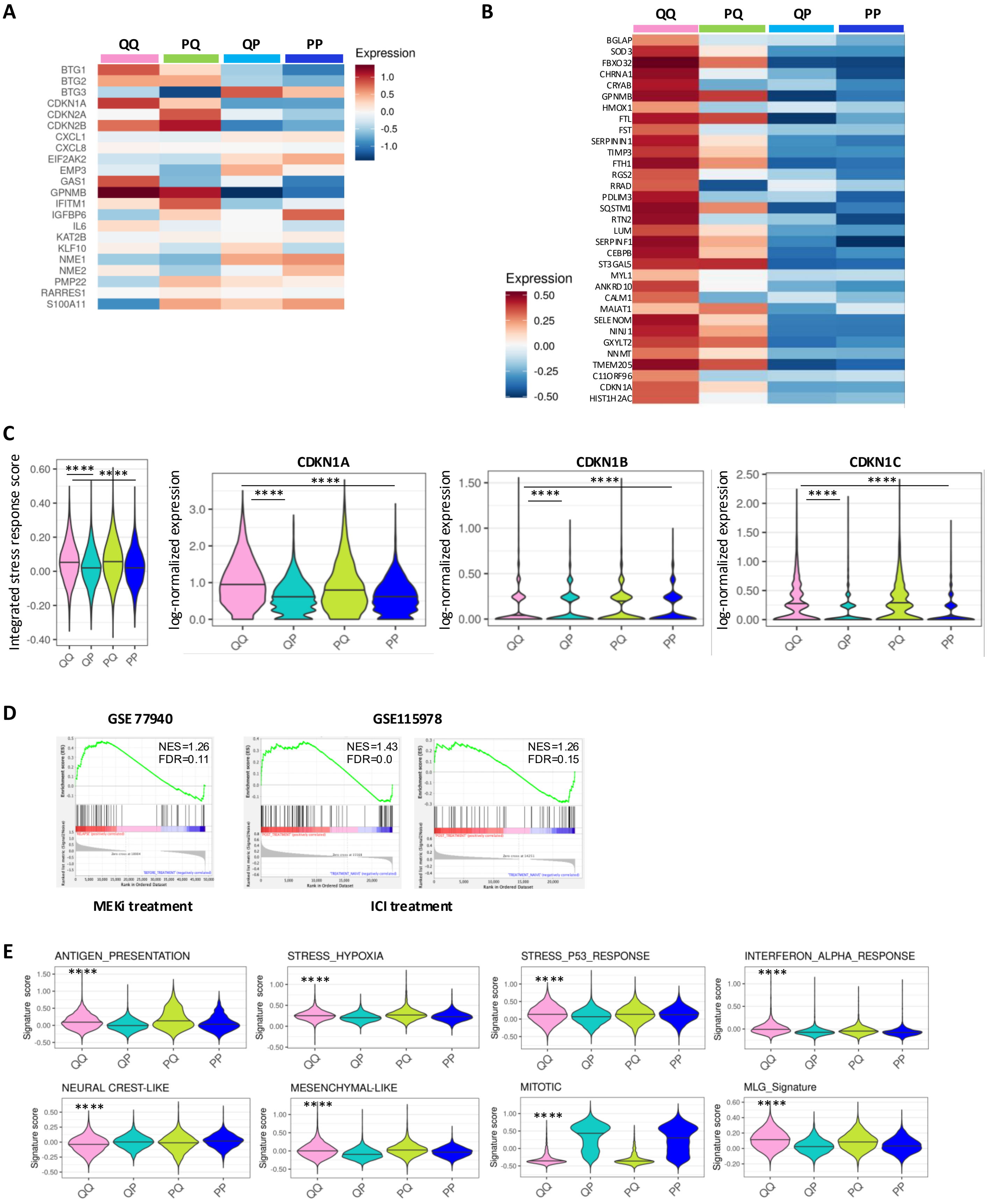

Appendix Figure S4. Characterization of QQ, PQ, QP and PP cell states.

A Heatmap of averaged expression of growth inhibitory gene set (GSEA cancer Module_488) in QQ, PQ, QP and PP cell states. **B** Heatmap of averaged expression of the top 33 genes of QQ signature (Dataset EV1) in QQ, PQ, QP and PP cell states. **C** Violin plot illustrating expression of integrated stress response genes and CDK inhibitor (CDKN1A, CDKN1B, CDKN1C) genes in PP, PQ, QP and QQ cell states. P-values were calculated using non-parametric Krustal-Wallis (Stress response genes ****P=2.25e-49; 1.02e-33; CDKN1A: ****P=6.23e-137; 7.73e-227; CDKN1B: ****P=6.94e-5; 8.69e-5; CDKN1C: ****P=6.26e-119; 8.12e-175). **D** GSEA enrichment analysis of QQ signature in RNA-seq dataset of RAF/MEKi treated patients before and after treatment (Data Ref: NCBI GEO dataset GSE77940) and in scRNA-seq (Data Ref: NCBI GEO GSE115978) dataset of patients before and after ICI treatment (FDR significant <0.25; Normalized Enriched Score NES indicated for each dataset). **E** Violin plot illustrating the expression of melanoma signatures from Pozniak (52) in QQ, QP, PQ and PP cell states. P-values were calculated using non-parametric Krustal-Wallis (QQvsPP and QQvsQP ****P<0.0001).

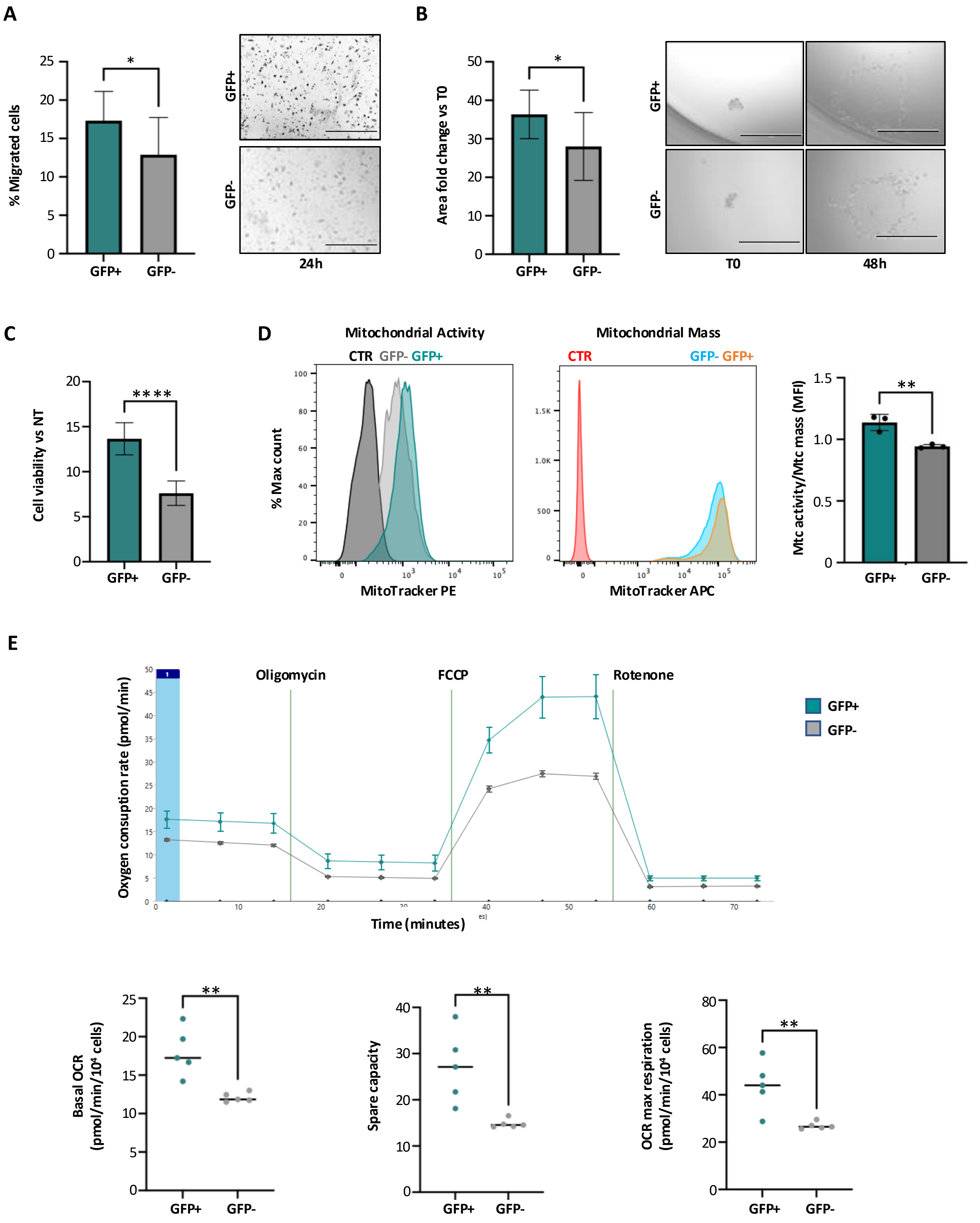

A
% Migrated cells
GFP+
GFP-
24h
B
Area fold change vs T0
T0
48h
C
Cell viability vs NT
D
Mitochondrial Activity
CTR GFP- GFP+
% Max count
MitoTracker PE
Mitochondrial Mass
CTR
GFP- GFP+
MitoTracker APC
Mtc activity/Mtc mass (MFI)
E
Oligomycin
FCCP
Rotenone
Oxygen consuption rate (pmol/min)
Time (minutes)
Basal OCR (pmol/min/10⁴ cells)
Spare capacity
OCR max respiration (pmol/min/10⁴ cells)

Appendix Figure S5. *In vitro* functional characterization of MM27 label-retaining cells.

A GFP+ and GFP- MM27 cell migration assessed by transwell migration assay at 24h. Representative EVOS microscopy images show migrated cells on the transwell outer surface. Data are mean+-SD (n=6 biological replicates). p-values are based on unpaired Student's t test (**P*=0.0316). Scale bar = 400µm. **B** Spheroid collagen invasion area in GFP+ and GFP- MM27 cells measured as fold change vs T0 (imaged daily by EVOS microscopy for 48h). Data are shown as the mean ± SD of different spheroids per group (n=8 technical replicates). Student t-test (**P*=0.0472). Representative images are shown. Scale bar = 1000µm. **C** Treatment of MM27 GFP+ and GFP- cells with 10nM trametinib for 72 hours *in vitro*. Cell viability assessed by CyQuant and normalized to DMSO control. Mean ± SD (n=9 technical replicates). p values are based on unpaired Student's t test (*****P*=4.74e-7). **D** GFP+ and GFP- MM27 cell mitochondrial activity assessed by flow cytometry analysis of MitoTracker Orange (25nM) stained cells normalized against mitochondrial mass measured in MitoTracker Deep Red stained cells. Data are shown as ratio of mitochondrial activity/mitochondrial mass mean fluorescence intensity ± SD (n=3 techical replicates). Student t-test (****P*=0.001). **E** Basal oxygen consumption rate (OCR), OCR spare capacity and OCR max respiration of GFP+ and GFP- MM27 cells assessed by Seahorse XF Cell Mito Stress. Data are mean ± SD (n=5 technical replicates). Student t-test (***P*=0.003; 0.008; 0.0072).

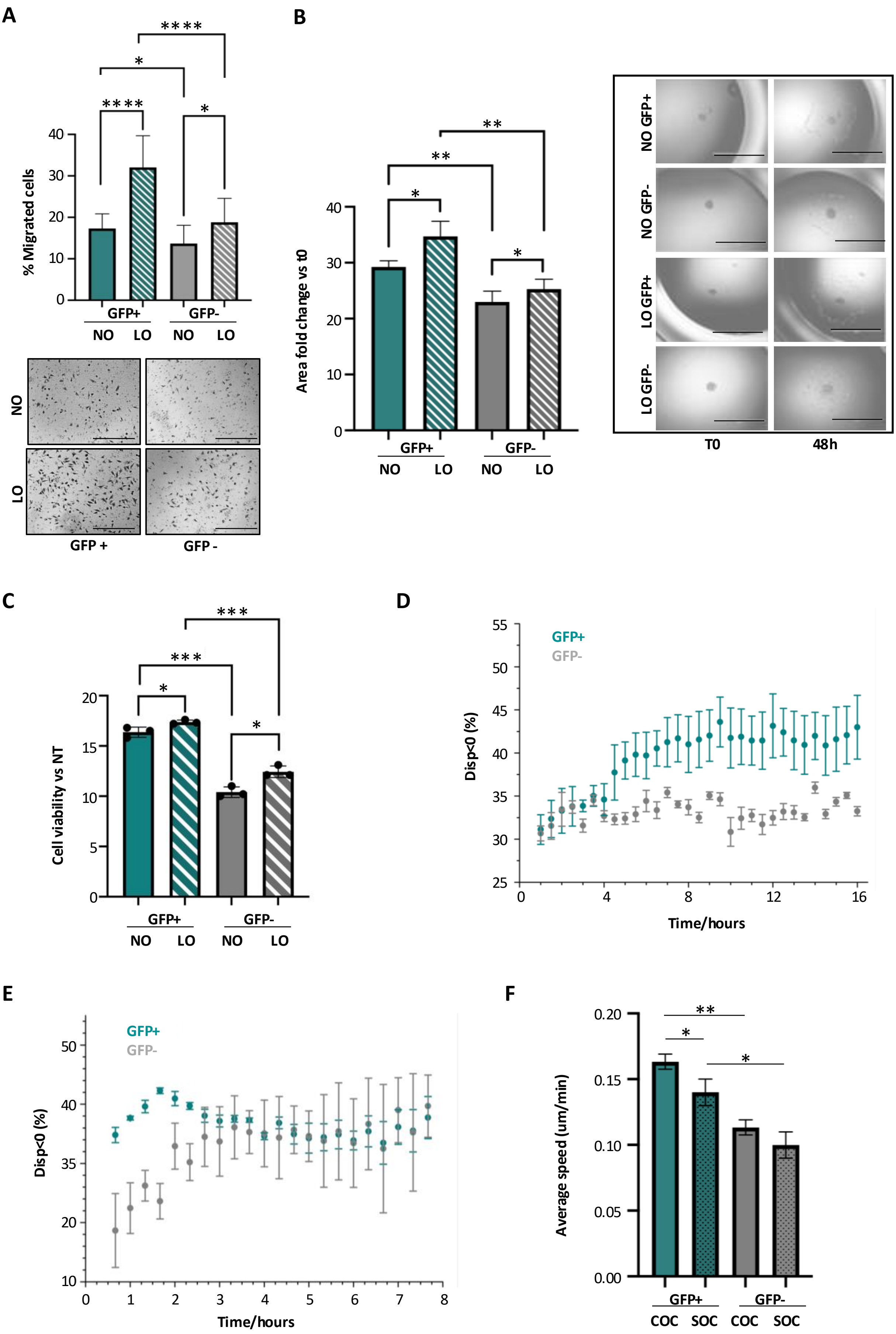
A
****
*
****
*
% Migrated cells
40
30
20
10
0
GFP+
GFP-
NO
LO
NO
LO
NO
LO
GFP +
GFP -
B
**
**
*
*
Area fold change vs t0
40
30
20
10
0
GFP+
GFP-
NO
LO
NO
LO
NO GFP+
NO GFP-
LO GFP+
LO GFP-
T0
48h
C
***
***
*
*
Cell viability vs NT
20
15
10
5
0
GFP+
GFP-
NO
LO
NO
LO
D
55
50
45
40
35
30
25
GFP+
GFP-
Disp<0 (%)
0
4
8
12
16
Time/hours
E
GFP+
GFP-
50
40
35
20
10
Disp<0 (%)
0
1
2
3
4
5
6
7
8
Time/hours
F
**
*
*
0.20
0.15
0.10
0.05
0.00
Average speed (um/min)
GFP+
GFP-
COC
SOC
COC
SOC

Appendix Figure S6. Impact of the hypoxic tumor microenvironment on MM27 label-retaining cells.

A GFP+ and GFP- cell migration assessed by transwell migration assay after 24h in normal (20%) and low (3%) oxygen conditions. Representative EVOS microscopy images show migrated cells on the transwell outer surface. Data are mean+-SD (n=15 technical replicates). p-values are based on unpaired Student's t test (* *P*=0.01086; 0.01954; ****P=2.36e-7;1.05e-5). Scale bar = 400µm. **B** Spheroid collagen invasion of GFP+ and GFP- cells in normal (20) and low (3%) oxygen conditions was measured as area of invasion fold change vs T0, imaged daily by EVOS microscopy for 48h. Data shown are the mean ± SD of n=5 different spheroids per group (technical replicates). Student t-test (* *P*=0.03193; 0.02056; ** *P*=0.00819; 0.007355). Representative images are shown. Scale bar = 1000µm. **C** *In vitro* 10nM trametinib treatment of GFP+ and GFP- cells in normal (20%) and low (3%) oxygen conditions for 72 hours. Cell viability was assessed by CyQuant and normalized to DMSO control (NT). Mean ± SD (n=3 biological replicates). p values are based on unpaired Student's t test (**P*=0.03526; 0.010; ****P*=0.00015; 0.00091). **D** Definition of displacement, *disp*, as function of distance of cell position at time *t*, *d(t)*, from zero oxygen level and % of disp<0 (% of cells migrating toward hypoxia) in GFP+ (green) and GFP- (gray) MM27 assessed during time-lapse of migration assay in chemical-controlled culture system (16 hours). Data are mean ± SD (n=3). Significance was assessed by one-way ANOVA (*P*=0.02298 and confidence level >95%). Number of trajectories analyzed (GFP+ n=1992; GFP- n=1568). **E** Percentage of cells moving toward hypoxia, *disp*<0, for GFP+ (green) and GFP- (gray) assessed during time-lapse of invasion assay in chemical-controlled culture system (8 hours). Data are mean ± SD (n=3 technical replicates). Significance was assessed by one-way ANOVA (*P*=0.03082 and confidence level >95%). Number of trajectories analyzed (GFP+ n=1136; GFP- n=481). **F** Average speed of GFP+ and GFP- cells in 3D collagen invasion assay analyzed in controlled (COC) and in standard (SOC) oxygen conditions. Data are mean ± SD (n=3 technical replicates). Student t test was applied to assess the significance (**P*=0.0131; 0.01473; ***P*=0.0056). Number of trajectories analyzed (GFP+ n=2179; GFP- n=1321).

Appendix Figure S7

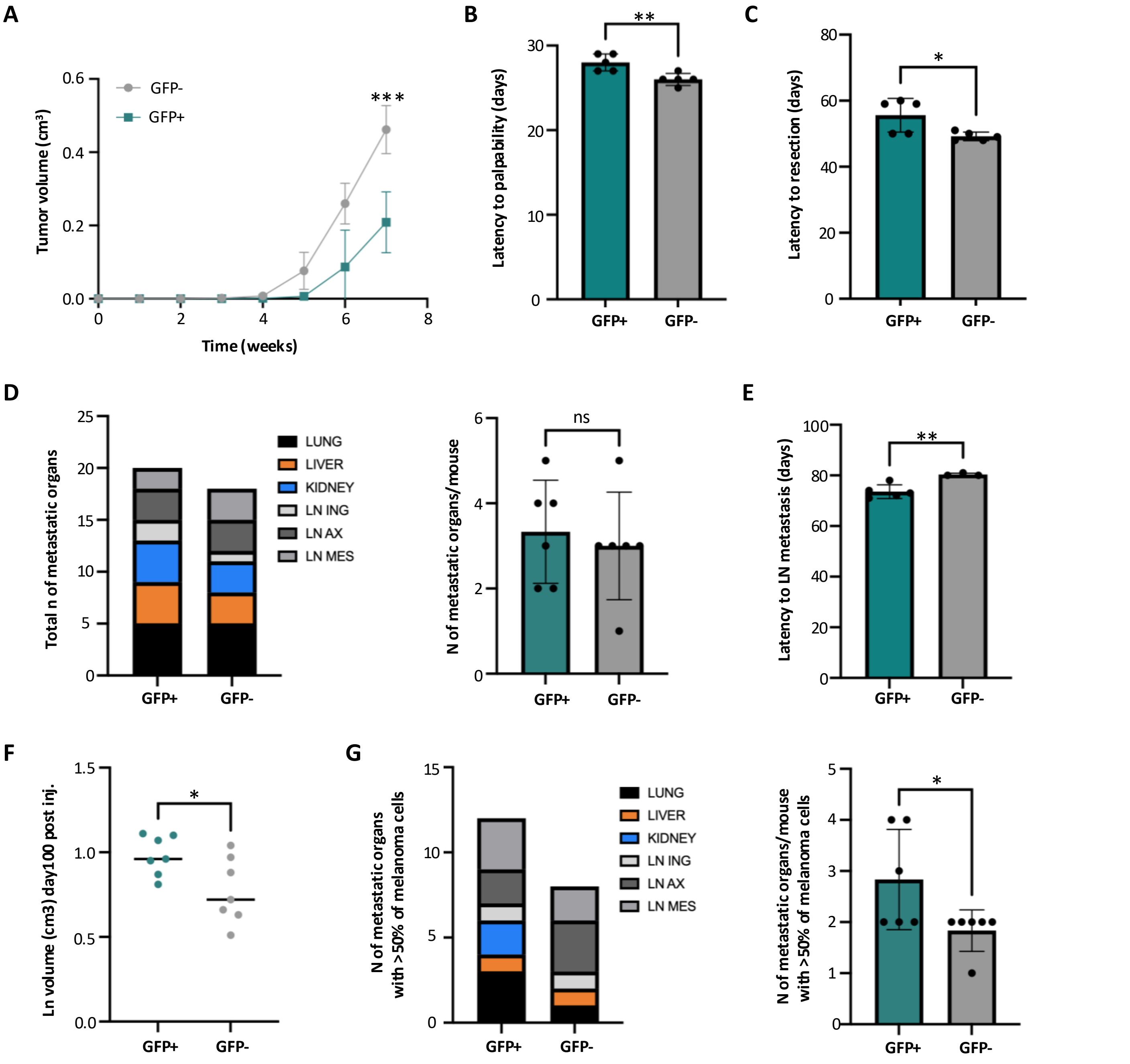

Appendix Figure S7. *In vivo* assessment of tumorigenic and invasive properties of MM27 GFP+ and GFP- populations in immunocompromised mice (n=5).

A Tumor growth at 6 weeks (GFP± n=5). **B** Latency to tumor palpability. **C** Latency to tumor resection (volume ~0.3cm^3) (GFP± n=5). **D** Total number of organs with detectable metastases (lung, liver, kidney, inguinal lymph node LN ING, axillary lymph node LN AX, mesenteric lymph node LN MES) and average number of organs with metastases *per* mouse. (GFP± n=6) **E** Latency to lymph node (LN) metastasis (days) (GFP± n=5). **F** LN volume at day100 post injection (GFP± n=7). **G** Total number of organs with at least 50% of melanoma cells and average number of organs with at least 50% of melanoma cells *per* mouse. (GFP± n=6). Data are mean ± SD. Student t test was applied to all the experiments to assess the significance (ns, not significant=0.651; *P=0.0269; 0.0317; 0.0442; **P=0.0065; 0.0061 ***P=0.0007).

Appendix Figure S8

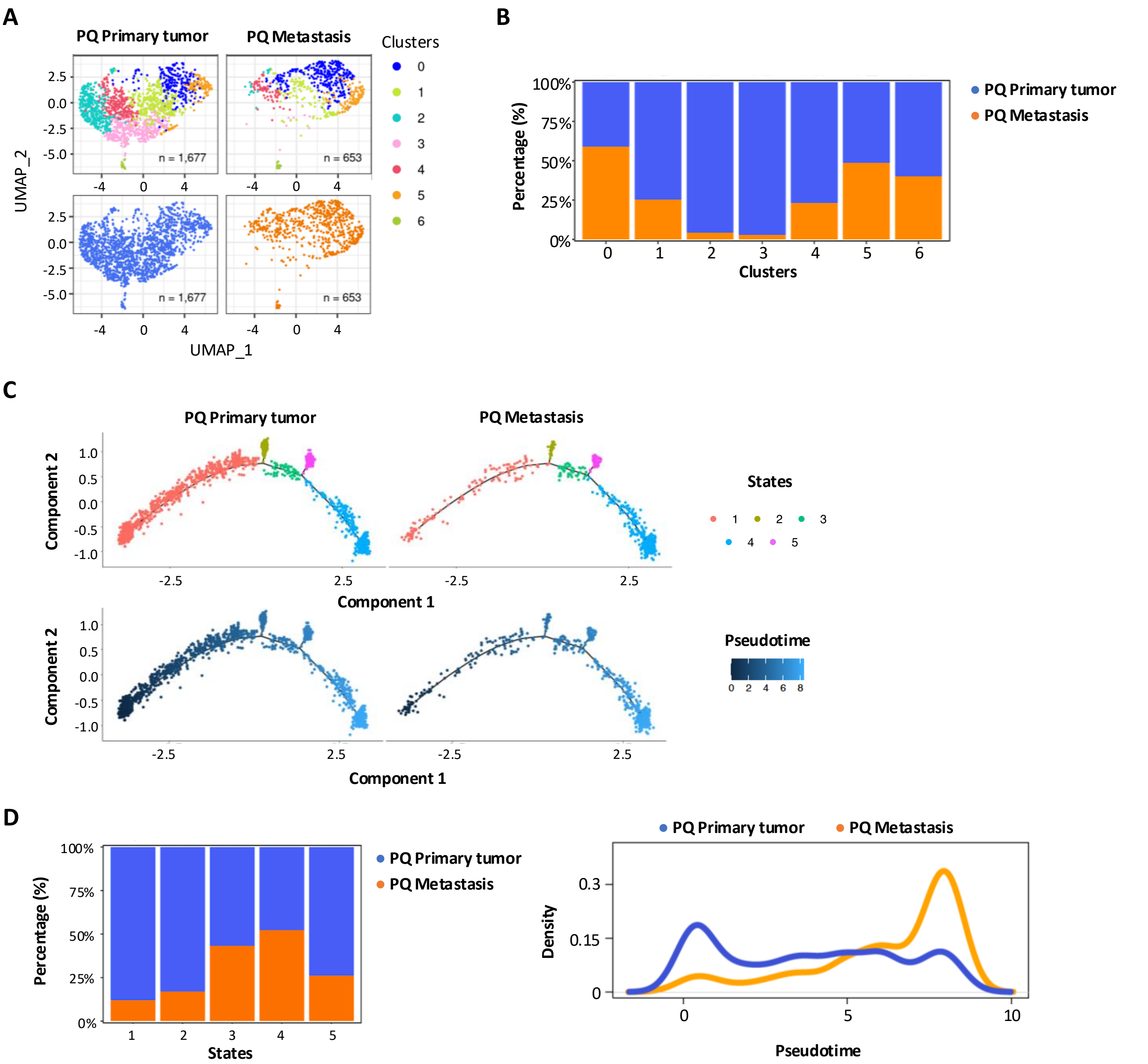

Appendix Figure S8. Analysis of transcriptional heterogeneity of MM13 PQ cells

A UMAP projection of the analysis of re-clustered quiescent PQ cells derived from primary tumor and metastatic samples. **B** Stack plot showing relative proportions of primary and metastatic quiescent PQ cells across Seurat clusters. **C** Trajectory analysis of primary and metastatic quiescent PQ cell states based on the expression of the top 20 variable genes. The trajectories are colored by state (upper panel) and pseudotime (lower panel). State 1 was selected as root state. **D** Stack plots (left) showing relative proportions of primary and metastatic quiescent PQ cells across pseudotime states; density plots (right) showing distribution of primary and metastatic PQ cells across along pseudotime.

Appendix Figure S9

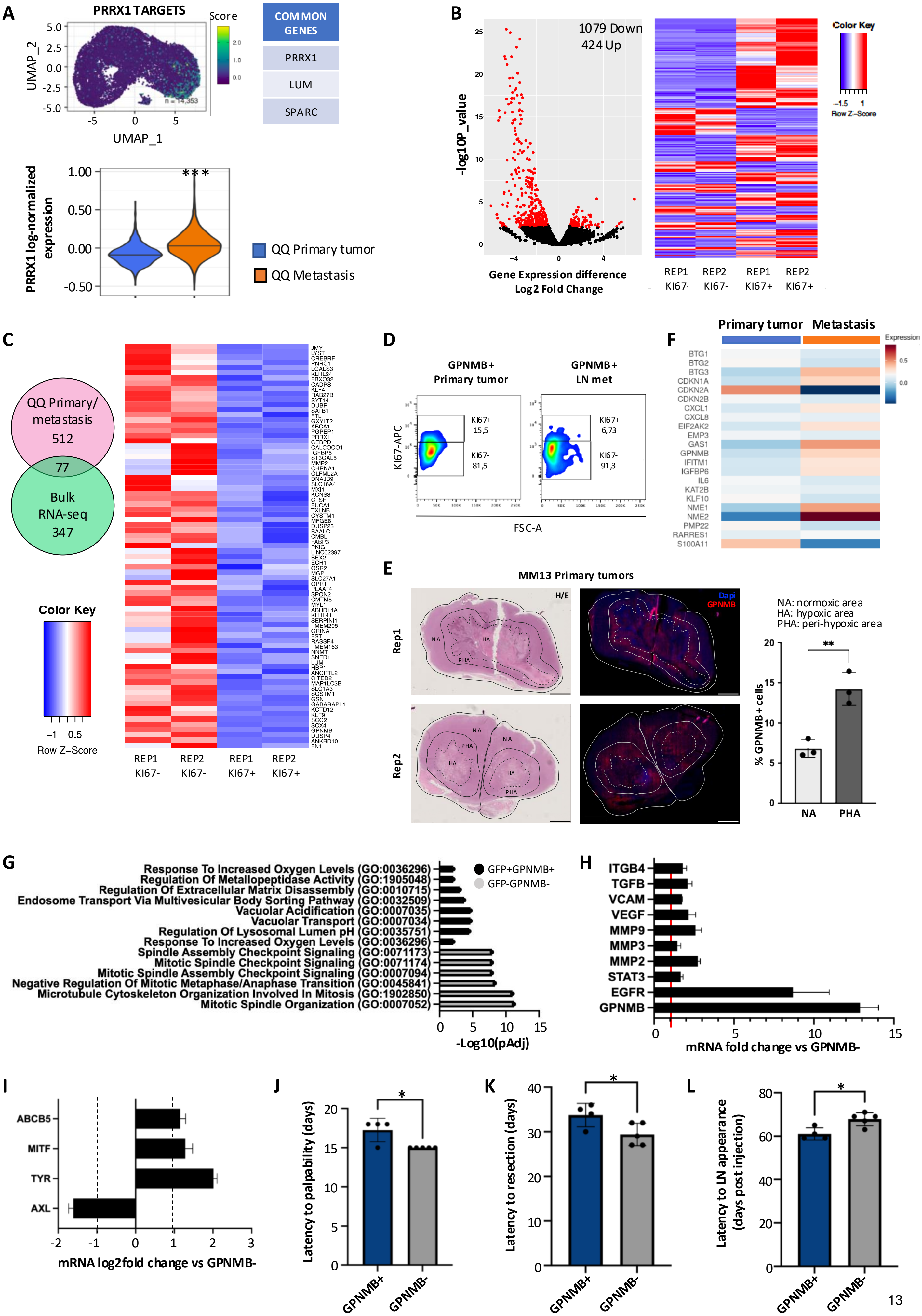

Appendix Figure S9. Transcriptomic profiling of QQ cell state identifies GPNMB as an actionable target within the quiescence signature.

A UMAP visualization (left) of MM13 melanoma dataset colored by the PRRX1 regulatory network from Karras et al (11); list of common genes among the 2 signatures (QQ/PQ MM13 signature and "mesenchymal like state" signature from Karras et al.); violin plot (right) showing expression of PRRX1 regulon in primary and metastatic QQ and PQ cell states. p-values were calculated using two-tailed Mann-Whitney U-test (*****P*=1.2e-36). **B** Volcano plot (left) and heatmap (right) of differentially expressed genes identified by bulk RNA-seq analysis of FACS sorted KI67- vs KI67+ populations. Two biological MM13 replicates were used for the experiment (REP1 and REP2). **C** Venn diagram and heatmap of common genes between the differentially expressed genes identified in B and the QQ signature. **D** FACS analysis of the percentage of KI67- and KI67+ cells within the MM13 primary tumor and lymph node metastasis GPNMB+ gated population. **E** Spatial distribution of GPNMB+ cells. FFPE MM13 primary tumors (biological replicates Rep1-Rep2) were stained with H/E (left) and for GPNMB/ Dapi by immunofluorescence (right); Scale bar = 2mm. Dashed lines indicate hypoxic areas (HA) and solid line identifies peri-hypoxic (PHA) and normoxic (NA) regions. The % of GPNMB+ cells is reported for NA and PHA areas (n=3 technical replicates; Student t test: ***P* = 0.0052). **F** Heatmap of averaged expression of growth inhibitory gene set (GSEA cancer Module_488) in QQ primary tumor and metastatic cells. **G** Enrichment pathway analysis (GO) of the GFP+GPNMB+ and GFP-GPNMB- populations. **H** QPCR of mRNA expression of genes involved in pathways triggered by GPNMB in GPNMB+ cells vs GPNMB- cells. **I** QPCR of mRNA expression of de-differentiation gene (AXL) and differentiation MITF downstream genes (TYR, MITF and ABCB5) in GPNMB+ cells vs GPNMB- cells. **J-K-L** *In vivo* assessment of tumorigenic and invasive properties of $2x10^4$ GPNMB+ and GPNMB- MM27 populations injected intradermically in NSG mice (Table S1). **J** latency to tumor palpability; **K** latency to tumor resection (volume ~0.3-0.4 cm^3); **L** latency to lymph node (LN) metastasis; Data are mean ± SD (GPNMB+ n=4; GPNMB- n=5). Student t test was applied to assess the significance (**P*=0.011; 0.0392; 0.0108).

Appendix Table S1. GFP+ and GFP- tumor latency to palpability, resection and metastasis formation

	Latency to palpability (days)	Latency to resection (days)	Latency to lymphnode metastasis (days)	Number of metastatic organs			
				Lung	Liver	Lymphnode	Kidney
MM13 GFP+							
M1	25	42	70	1	1	1	1
M2	25	50	70	1	1	1	1
M3	25	42	77	1	1	1	0
M4	27	50	70	1	0	1	1
M5	25	50	77	1	1	1	1
MM13 GFP-							
M1	22	40	84	0	0	1	1
M2	22	44	/	1	1	1	0
M3	22	39	79	1	0	1	1
M4	26	44	/	1	0	1	1
M5	22	40	79	1	1	1	1
MM27 GFP+							
M1	27	50	73	1	1	1	2
M2	27	59	74	1	1	1	1
M3	28	59	72	1	1	1	1
M4	29	60	73	1	0	1	2
M5	29	50	78	1	1	0	1
MM27 GFP-							
M1	27	51	80	1	1	1	2
M2	26	50	81	1	0	1	1
M3	25	49	80	1	1	0	1
M4	26	48	/	1	1	0	1
M5	26	48	/	1	0	1	1

Appendix Table S2. GPNMB+ and GPNMB- tumor latency to palpability, resection and metastasis formation

	Latency to palpability (days)	Latency to resection (days)	Latency to lymphnode metastasis (days)
MM13 GPNMB+			
M1	24	43	54
M2	24	34	57
M3	21	43	52
M4	24	34	57
M5	24	35	59
M6	21	43	63
MM13 GFNMB-			
M1	17	29	59
M2	24	31	65
M3	17	29	65
M4	17	35	69
M5	17	28	67
M6	24	40	72
MM27 GPNMB+			
M1	15	30	59
M2	18	35	61
M3	18	34	65
M4	18	36	59
M5			
MM27 GPNMB-			
M1	15	32	63
M2	15	29	67
M3	15	32	69
M4	15	27	69
M5	15	27	71

Appendix Table S3. GV treatment in MM13 PDX

	Latency to resection (days)	Lung	Liver	Lymphnode	Kidney
MM13 (vehicle)					
M1	28	1	1	1	0
M2	27	1	1	1	0
M3	27	1	1	1	0
M4	28	1	1	1	0
M5	35	1	1	1	0
M6	32	1	1	1	0
M7	33	1	1	1	0
MM13 (GV)					
M1	37	1	1	1	0
M2	40	1	1	0	0
M3	39	0	1	0	0
M4	28	0	0	0	0
M5	28	0	0	0	0
M6	40	0	0	0	0
