# Supplementary figures and images for: GPNMB marks a quiescent cell population in melanoma and promotes metastasis formation
Source: EMBO Rep. 2025 Jun 17;26(15):3804–30. doi: 10.1038/s44319-025-00501-w (PMC12332017; doi:10.1038/s44319-025-00501-w)

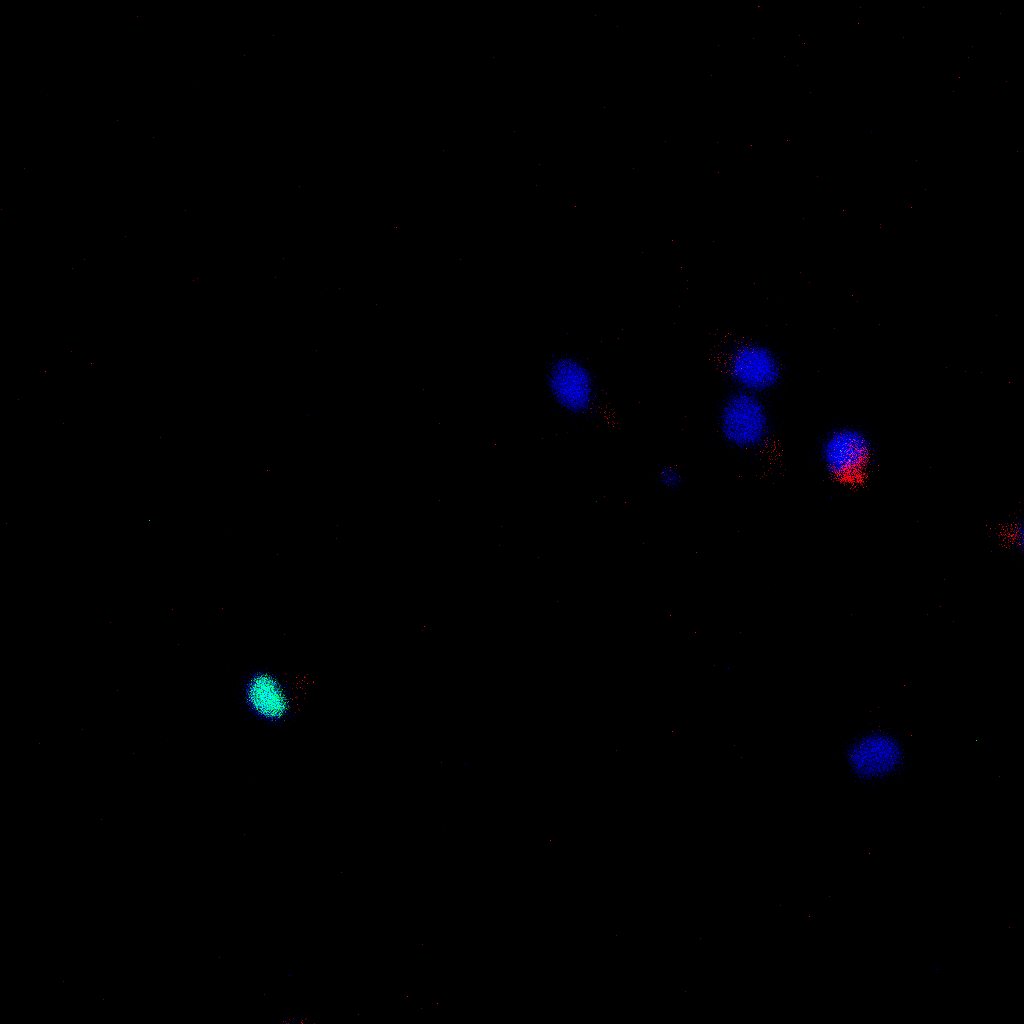

Supplement: Supplementary file 5 — Source data Fig. 1 [file 44319_2025_501_MOESM5_ESM.zip › Figure1/1D/IMAGES/Images for quantification/Image E.tif]

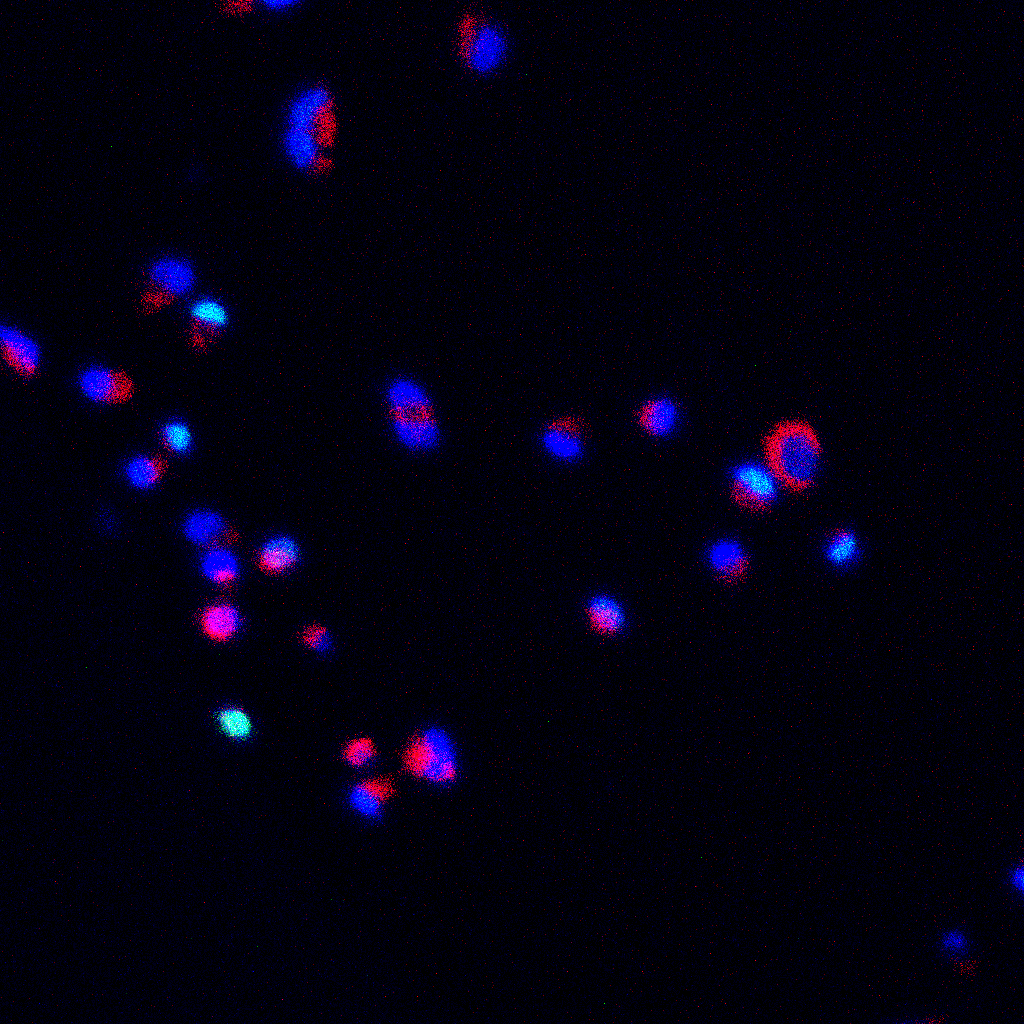

Supplement: Supplementary file 5 — Source data Fig. 1 [file 44319_2025_501_MOESM5_ESM.zip › Figure1/1D/IMAGES/Images for quantification/Image D.tif]

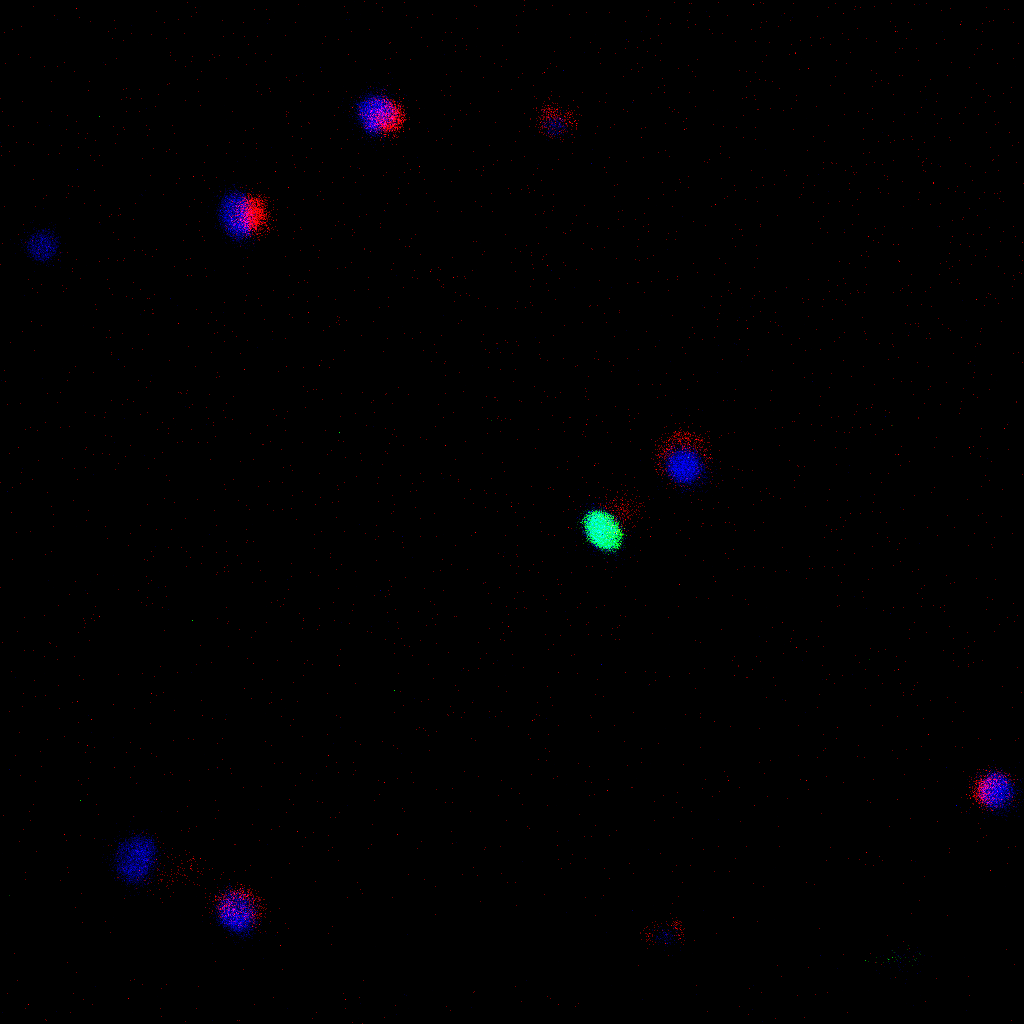

Supplement: Supplementary file 5 — Source data Fig. 1 [file 44319_2025_501_MOESM5_ESM.zip › Figure1/1D/IMAGES/Images for quantification/Image A.tif]

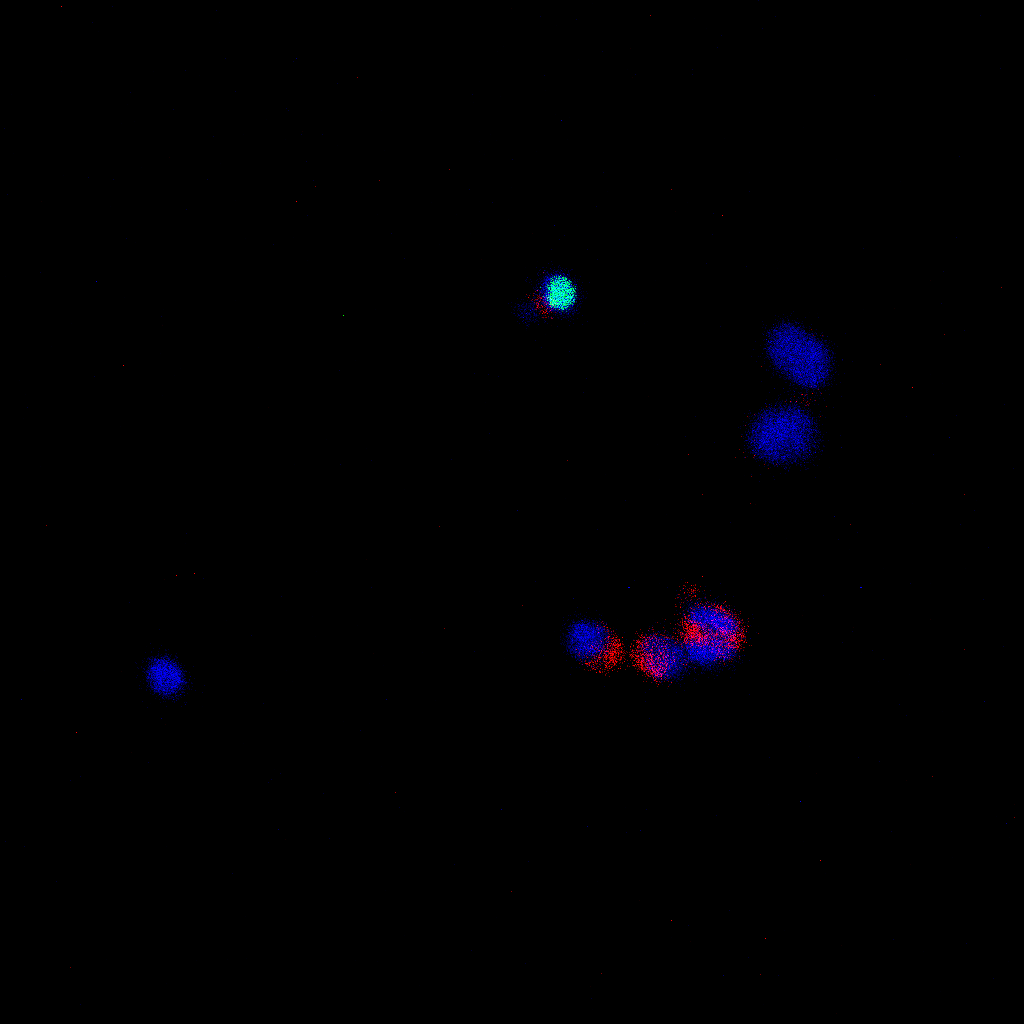

Supplement: Supplementary file 5 — Source data Fig. 1 [file 44319_2025_501_MOESM5_ESM.zip › Figure1/1D/IMAGES/Images for quantification/Image C.tif]

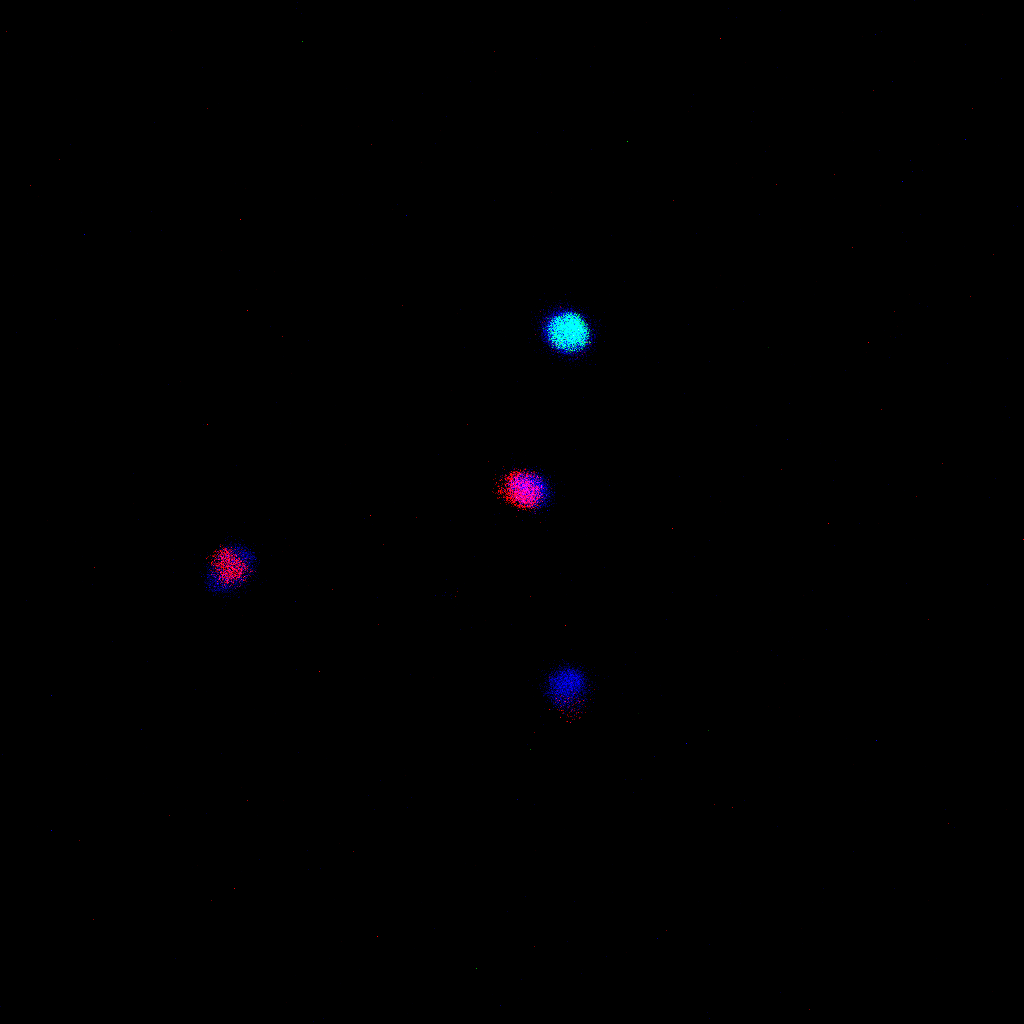

Supplement: Supplementary file 5 — Source data Fig. 1 [file 44319_2025_501_MOESM5_ESM.zip › Figure1/1D/IMAGES/Images for quantification/Image B.tif]

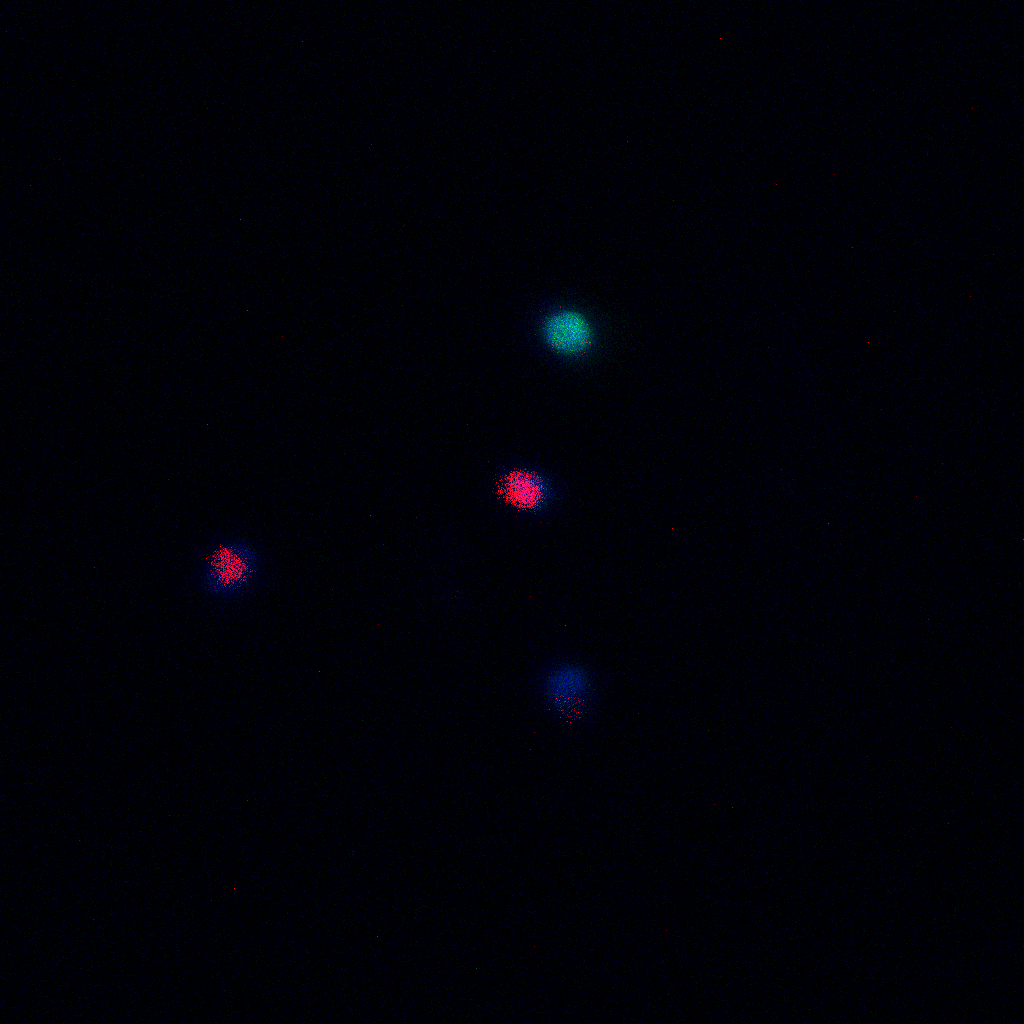

Supplement: Supplementary file 5 — Source data Fig. 1 [file 44319_2025_501_MOESM5_ESM.zip › Figure1/1D/IMAGES/Representative Image Figure 1D/MAX_Composite (RGB)7.tif]

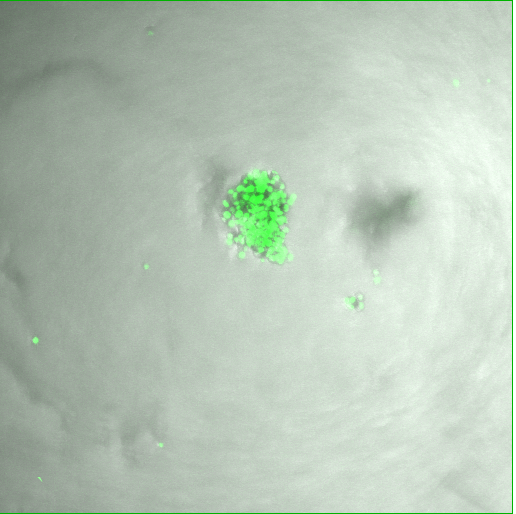

Supplement: Supplementary file 6 — Source data Fig. 4 [file 44319_2025_501_MOESM6_ESM.zip › Figure4/4B/REPRESENTATIVE IMAGES/MM13 GFP+ well5 T0.png]

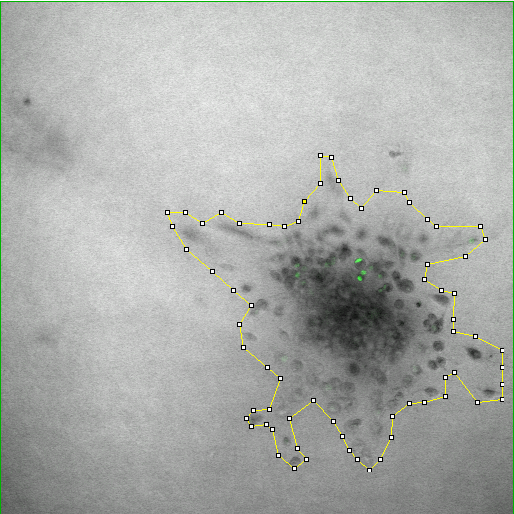

Supplement: Supplementary file 6 — Source data Fig. 4 [file 44319_2025_501_MOESM6_ESM.zip › Figure4/4B/REPRESENTATIVE IMAGES/MM13 GFP- well1 T48h.png]

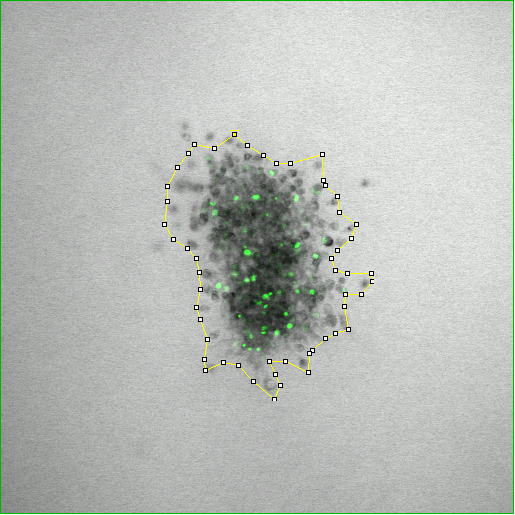

Supplement: Supplementary file 6 — Source data Fig. 4 [file 44319_2025_501_MOESM6_ESM.zip › Figure4/4B/REPRESENTATIVE IMAGES/MM13 GFP- well4 T24h.png]

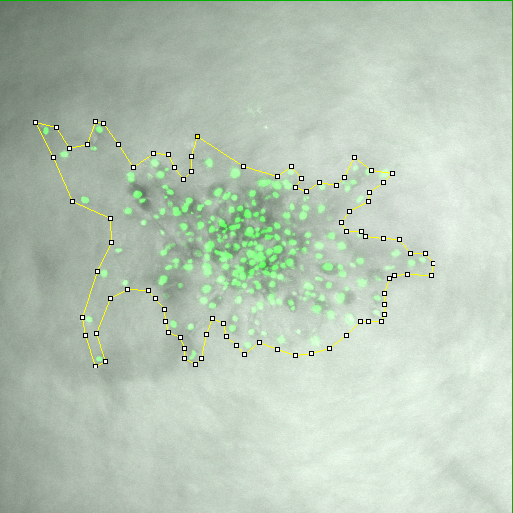

Supplement: Supplementary file 6 — Source data Fig. 4 [file 44319_2025_501_MOESM6_ESM.zip › Figure4/4B/REPRESENTATIVE IMAGES/MM13 GFP+ well5 T48h.png]

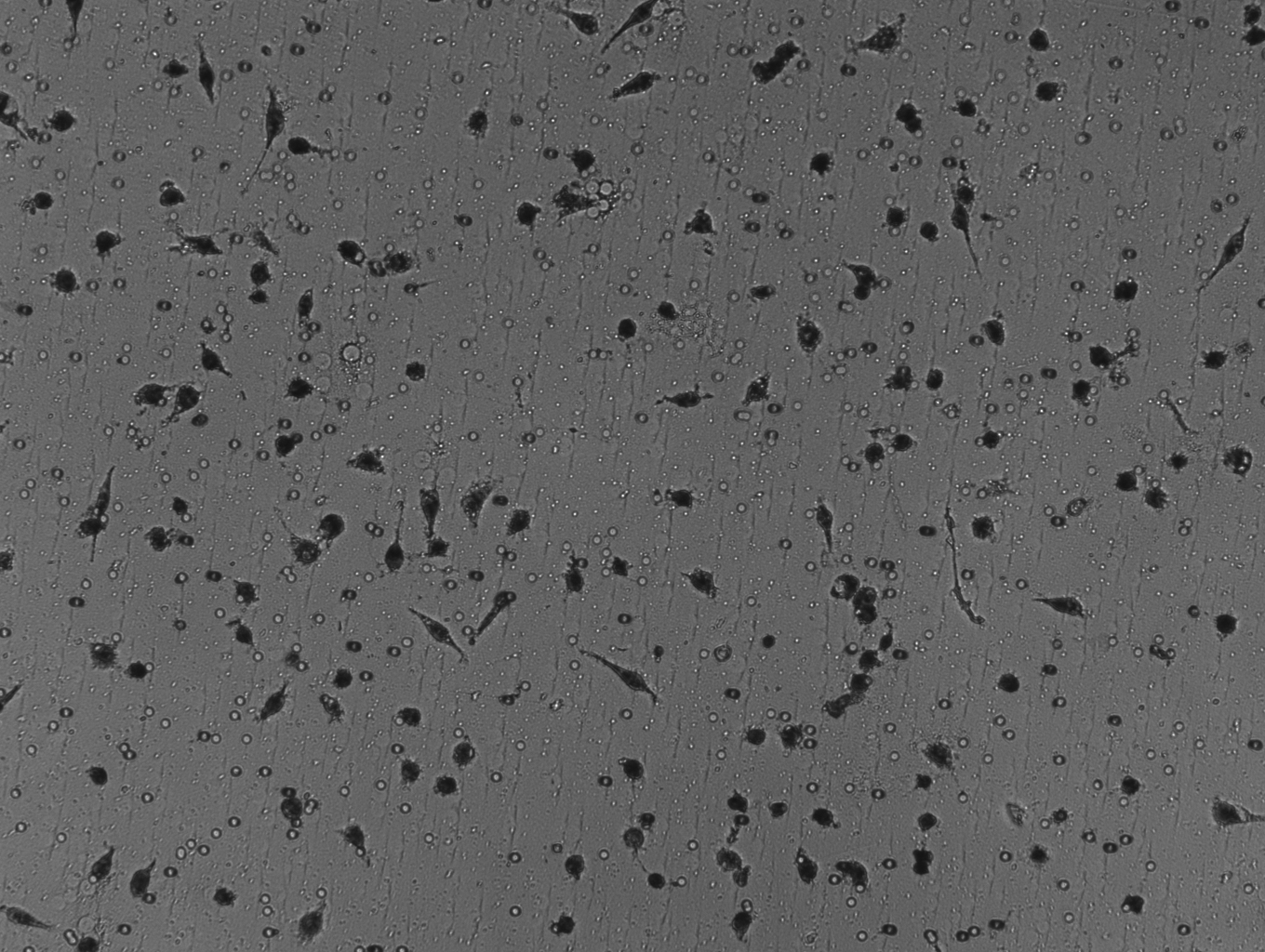

Supplement: Supplementary file 6 — Source data Fig. 4 [file 44319_2025_501_MOESM6_ESM.zip › Figure4/4A/ REPRESENTATIVE IMAGES/MM13 GFP neg well2 pic3 10x.tif]

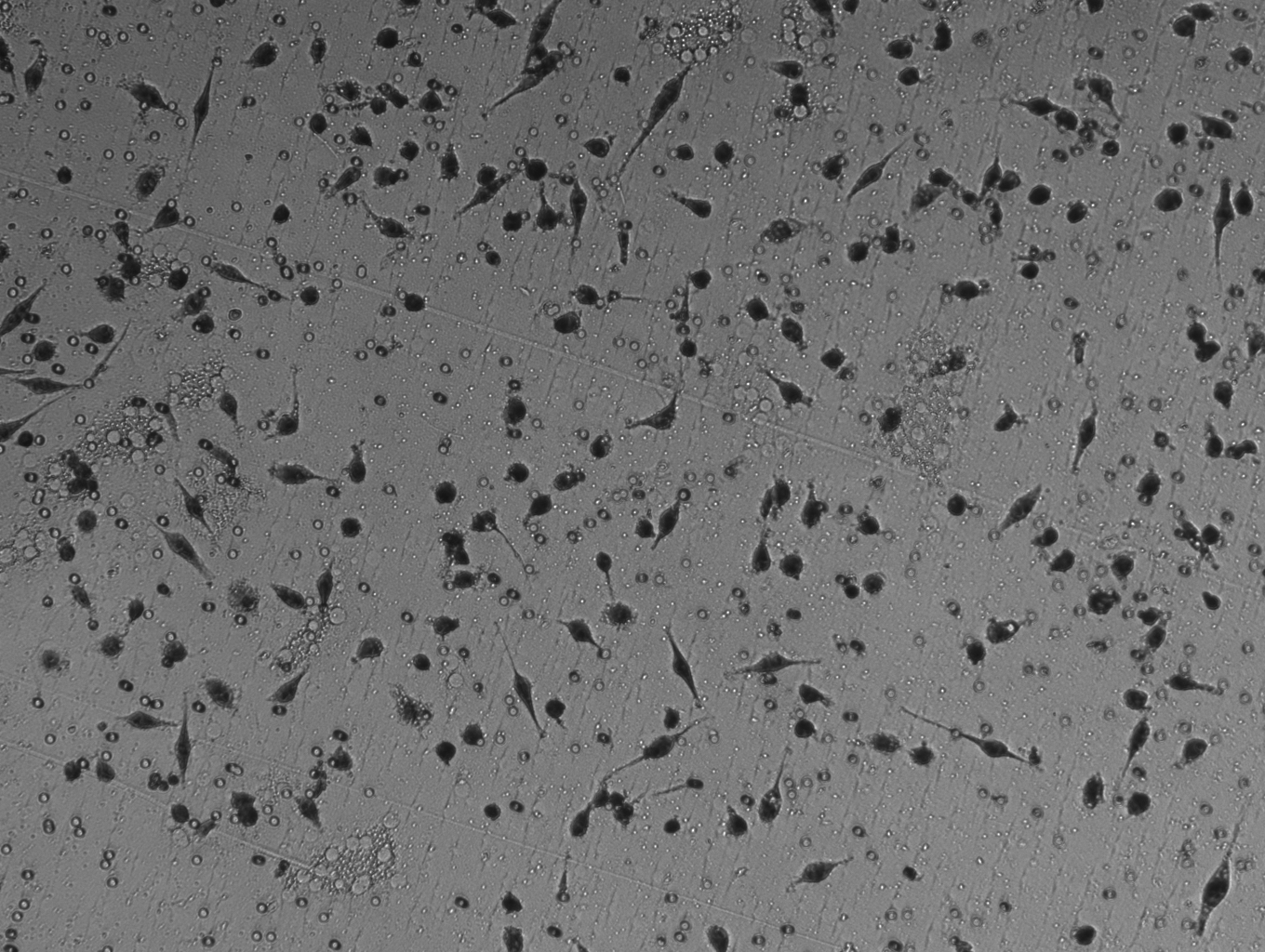

Supplement: Supplementary file 6 — Source data Fig. 4 [file 44319_2025_501_MOESM6_ESM.zip › Figure4/4A/ REPRESENTATIVE IMAGES/MM13 GFP pos well2 pic8 10x.tif]

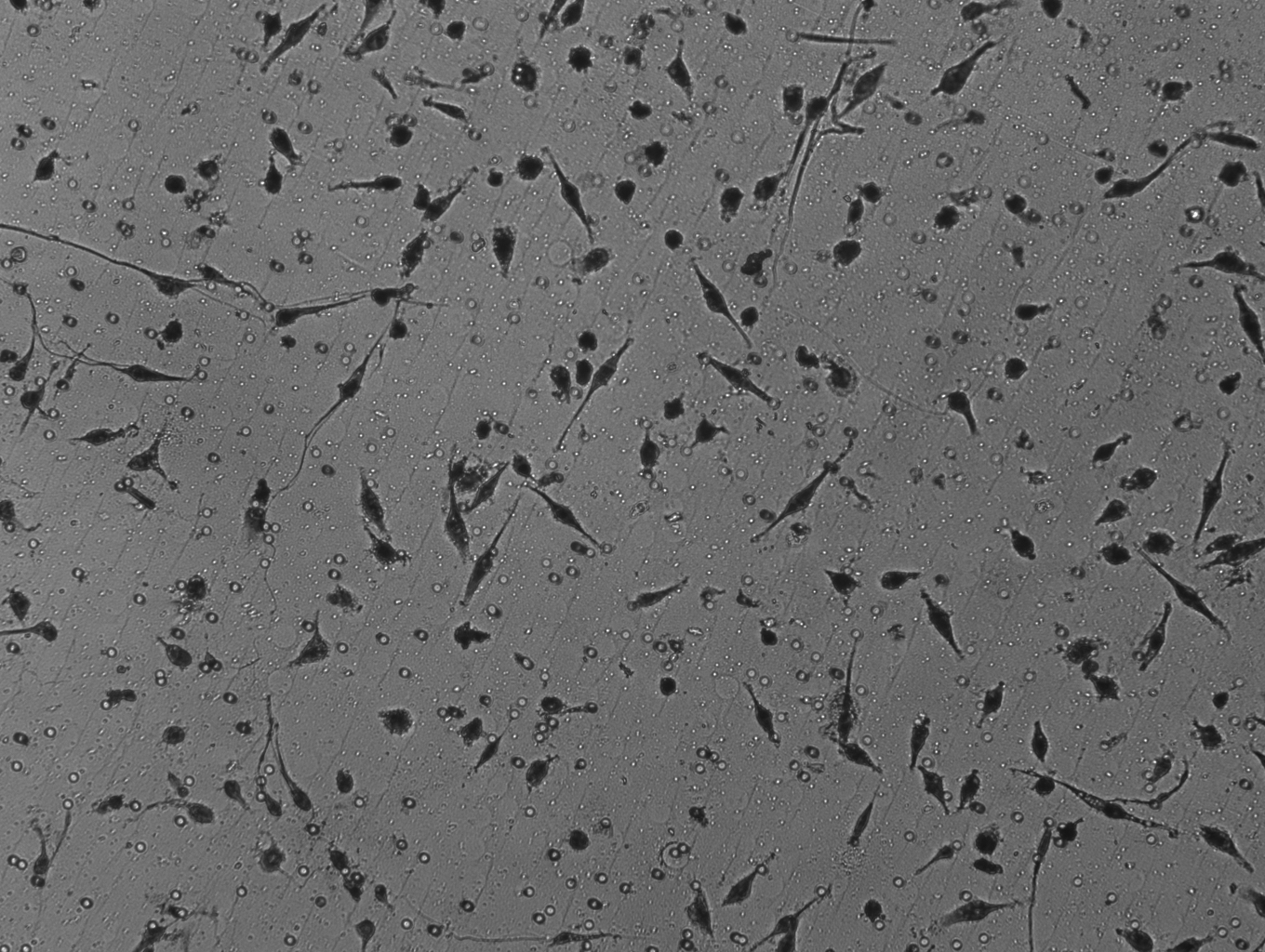

Supplement: Supplementary file 7 — Source data Fig. 5 [file 44319_2025_501_MOESM7_ESM.zip › Figure5/5A/Representative Images/MM13 GFP+ LO.tif]

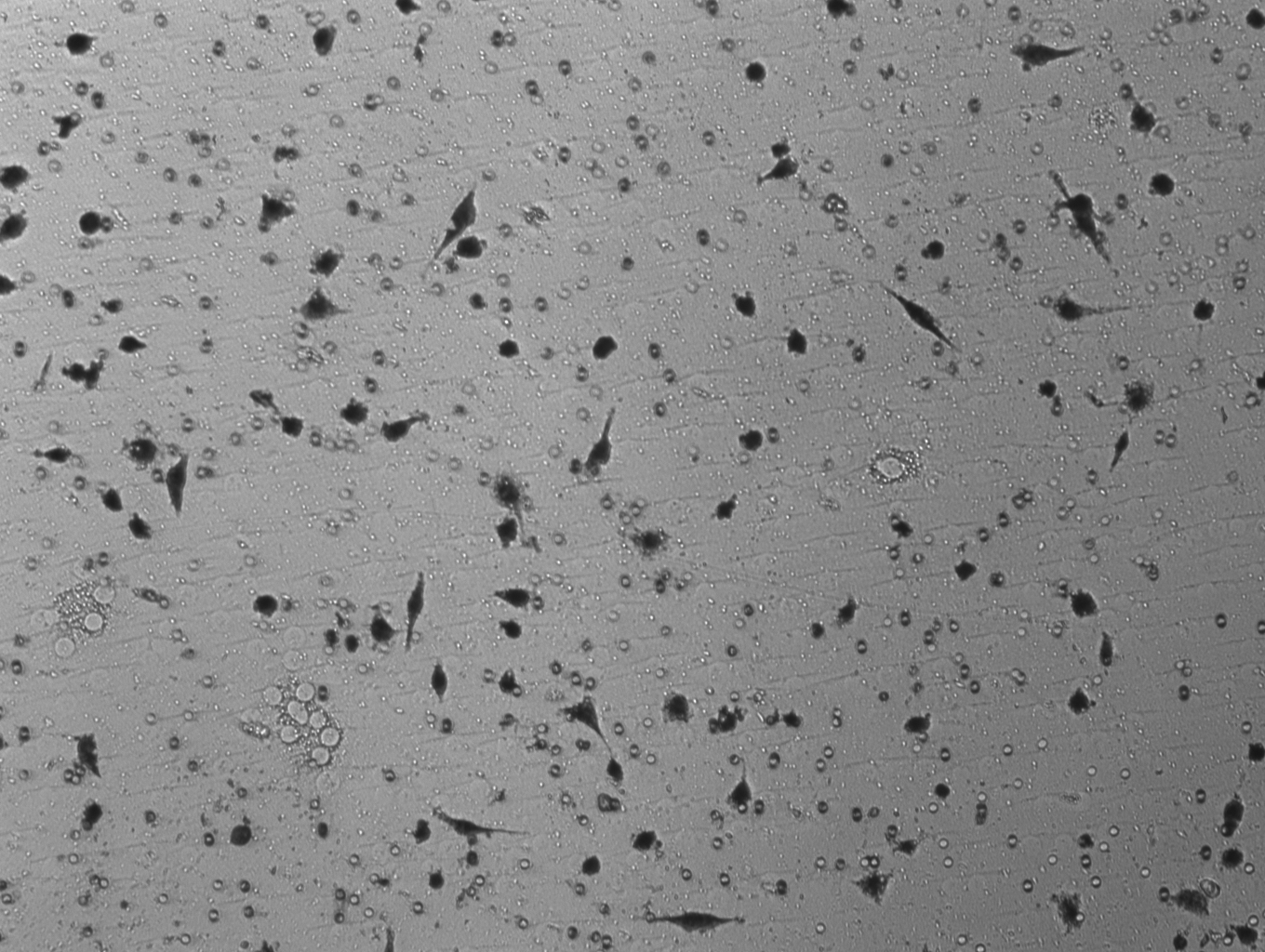

Supplement: Supplementary file 7 — Source data Fig. 5 [file 44319_2025_501_MOESM7_ESM.zip › Figure5/5A/Representative Images/MM13 GFP- LO.tif]

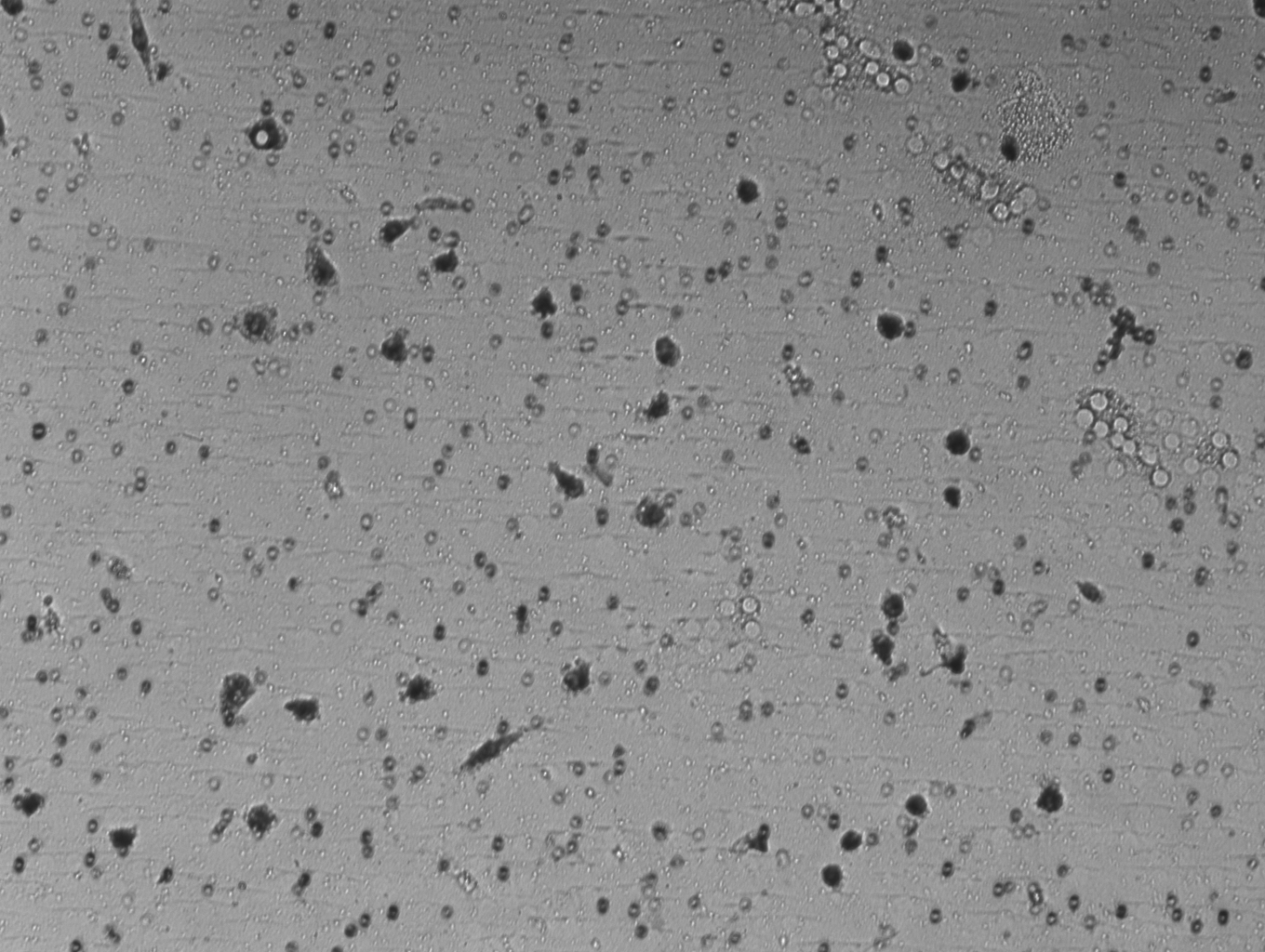

Supplement: Supplementary file 7 — Source data Fig. 5 [file 44319_2025_501_MOESM7_ESM.zip › Figure5/5A/Representative Images/MM13 GFP- NO.tif]

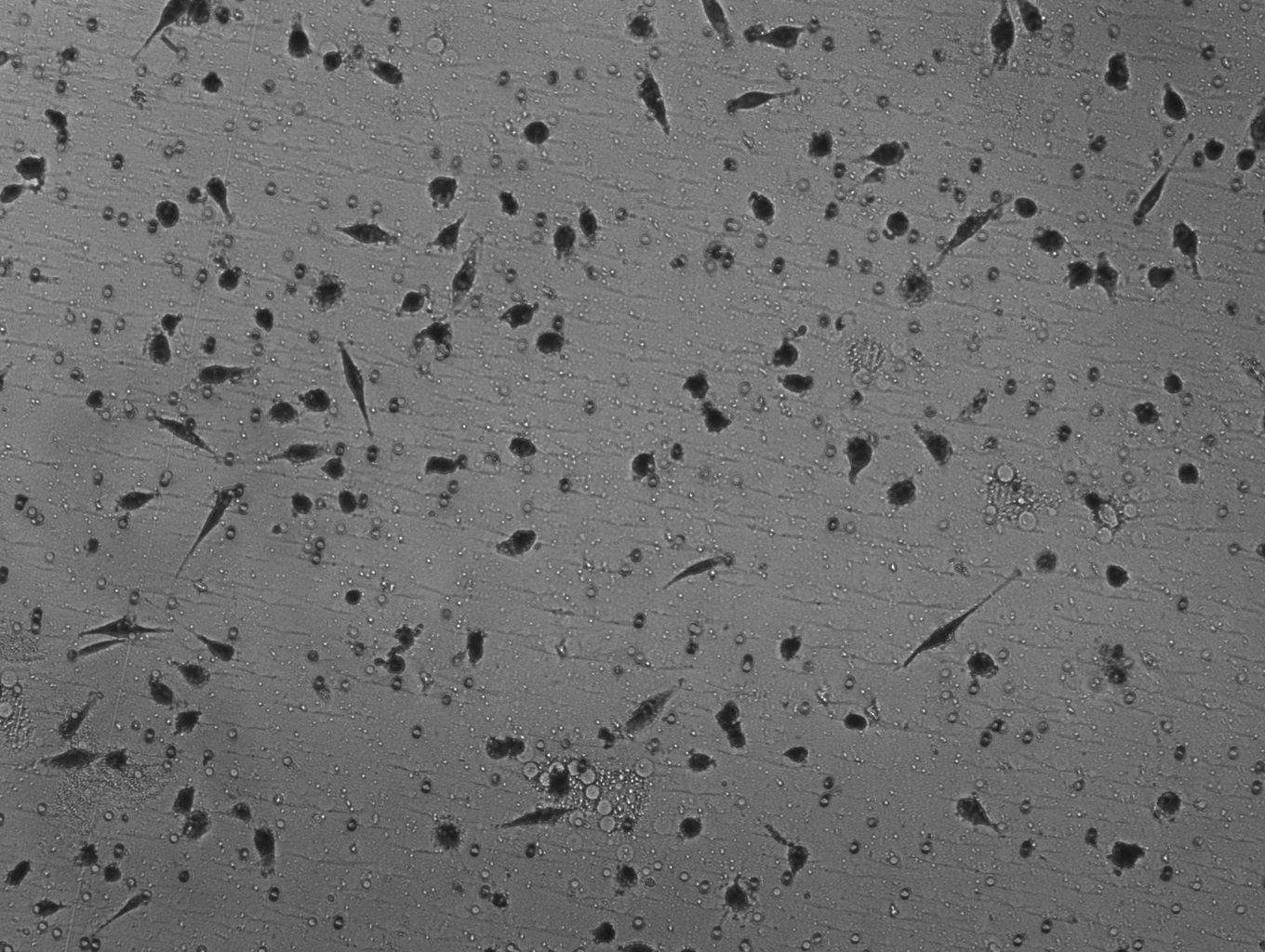

Supplement: Supplementary file 7 — Source data Fig. 5 [file 44319_2025_501_MOESM7_ESM.zip › Figure5/5A/Representative Images/MM13 GFP+ NO.tif]

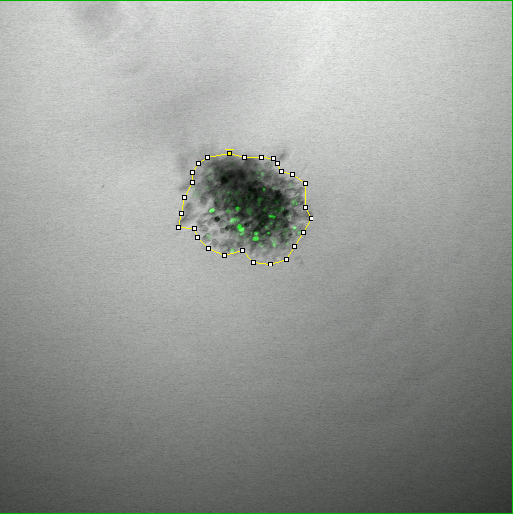

Supplement: Supplementary file 7 — Source data Fig. 5 [file 44319_2025_501_MOESM7_ESM.zip › Figure5/5B/Representative Images/Image LO GFP- T0-T48h/LO GFP- T0 .png]

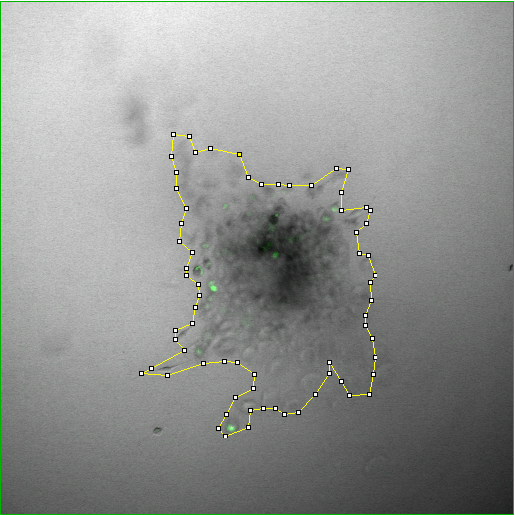

Supplement: Supplementary file 7 — Source data Fig. 5 [file 44319_2025_501_MOESM7_ESM.zip › Figure5/5B/Representative Images/Image LO GFP- T0-T48h/LO GFP- T48.png]

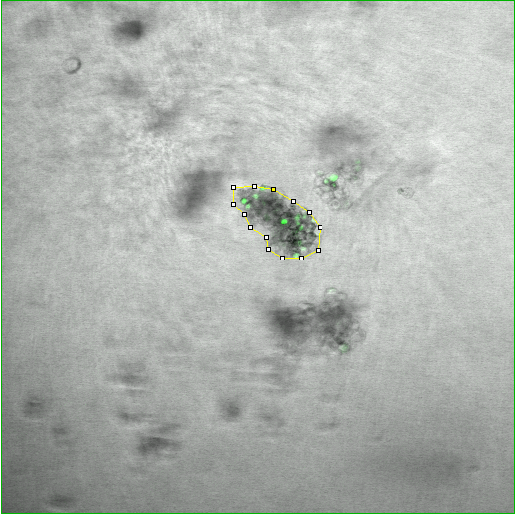

Supplement: Supplementary file 7 — Source data Fig. 5 [file 44319_2025_501_MOESM7_ESM.zip › Figure5/5B/Representative Images/Image NO GFP- T0-T48h/NO GFP- T0.png]

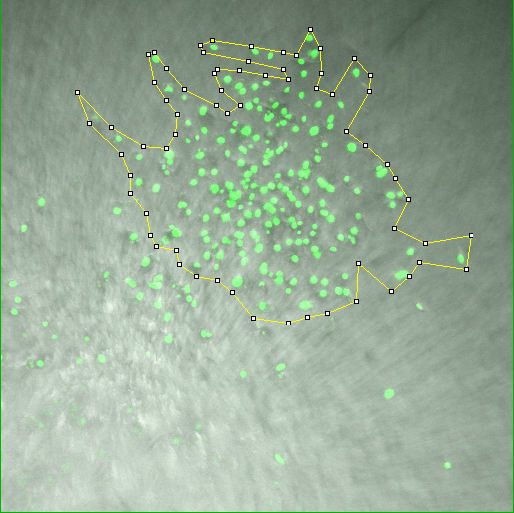

Supplement: Supplementary file 7 — Source data Fig. 5 [file 44319_2025_501_MOESM7_ESM.zip › Figure5/5B/Representative Images/Image LO GFP+ T0-T48h/LO GFP+ T48h.png]

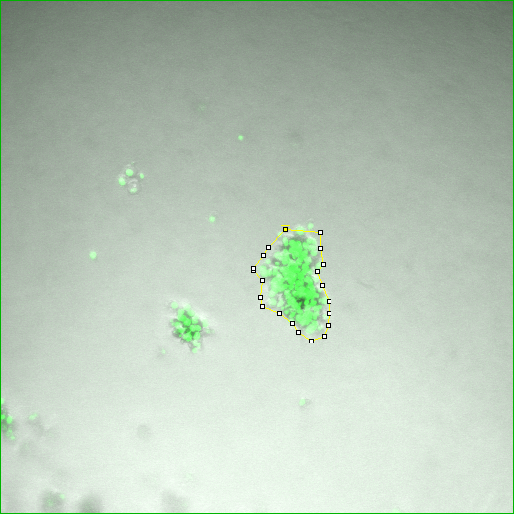

Supplement: Supplementary file 7 — Source data Fig. 5 [file 44319_2025_501_MOESM7_ESM.zip › Figure5/5B/Representative Images/Image LO GFP+ T0-T48h/LO GFP+ T0.png]
